# Development and application of oncolytic viruses as the nemesis of tumor cells

**DOI:** 10.3389/fmicb.2023.1188526

**Published:** 2023-06-12

**Authors:** Xiao Zhu, Chenyang Fan, Zhuolong Xiong, Mingwei Chen, Zesong Li, Tao Tao, Xiuqing Liu

**Affiliations:** ^1^Zhejiang Provincial People's Hospital Affiliated to Hangzhou Medical College, Hangzhou Medical College, Hangzhou, China; ^2^The Marine Biomedical Research Institute, Guangdong Medical University, Zhanjiang, China; ^3^Department of Biological and Chemical Sciences, New York Institute of Technology—Manhattan Campus, New York, NY, United States; ^4^Department of Clinical Medicine, Medicine and Technology, School of Zunyi Medical University, Zunyi, China; ^5^Guangdong Provincial Key Laboratory of Systems Biology and Synthetic Biology for Urogenital Tumors, Shenzhen Key Laboratory of Genitourinary Tumor, Department of Urology, The First Affiliated Hospital of Shenzhen University, Shenzhen Second People's Hospital(Shenzhen Institute of Translational Medicine), Shenzhen, China; ^6^Department of Gastroenterology, Zibo Central Hospital, Zibo, China; ^7^Department of Clinical Laboratory, Institute of Translational Medicine, The First Affiliated Hospital of Shenzhen University, Shenzhen Second People’s Hospital, Shenzhen, China

**Keywords:** anti-tumor immune response, oncolytic virus, tumor cells, PD-1, adenovirus, herpes simplex virus

## Abstract

Viruses and tumors are two pathologies that negatively impact human health, but what occurs when a virus encounters a tumor? A global consensus among cancer patients suggests that surgical resection, chemotherapy, radiotherapy, and other methods are the primary means to combat cancer. However, with the innovation and development of biomedical technology, tumor biotherapy (immunotherapy, molecular targeted therapy, gene therapy, oncolytic virus therapy, etc.) has emerged as an alternative treatment for malignant tumors. Oncolytic viruses possess numerous anti-tumor properties, such as directly lysing tumor cells, activating anti-tumor immune responses, and improving the tumor microenvironment. Compared to traditional immunotherapy, oncolytic virus therapy offers advantages including high killing efficiency, precise targeting, and minimal side effects. Although oncolytic virus (OV) therapy was introduced as a novel approach to tumor treatment in the 19th century, its efficacy was suboptimal, limiting its widespread application. However, since the U.S. Food and Drug Administration (FDA) approved the first OV therapy drug, T-VEC, in 2015, interest in OV has grown significantly. In recent years, oncolytic virus therapy has shown increasingly promising application prospects and has become a major research focus in the field of cancer treatment. This article reviews the development, classification, and research progress of oncolytic viruses, as well as their mechanisms of action, therapeutic methods, and routes of administration.

## 1. Introduction

In recent years, significant advances in cancer genomics and the application of various therapeutic interventions, such as chemotherapy, radiation therapy, and immunotherapy, have led to remarkable improvements in the prognosis of cancer patients. However, these treatments still have substantial limitations, including (1) serious side effects associated with chemotherapy and radiation therapy, (2) development of drug resistance in tumor cells, and (3) suboptimal efficacy of immunotherapy in severe and highly immunosuppressed diseases (Chiocca and Rabkin, 2014; Zhu et al., 2022; Tripodi et al., 2023). Therefore, the development of a novel therapeutic approach is necessary, which should avoid adverse effects on normal tissue cells while exhibiting high specificity for tumor cells. Oncolytic viruses (OVs) are natural or genetically modified drugs that possess a characteristic of elevated sensitivity to innate antiviral signaling and dependence on tumor signaling pathways, allowing for targeted infection and intracellular proliferation within tumor cells, triggering both innate and adaptive immune responses in the host, ultimately resulting in tumor cell death (Chiocca and Rabkin, 2014; Ghasemi Darestani et al., 2023; Malhotra and Kim, 2023). This property renders oncolytic viruses a promising therapeutic approach for cancer treatment. Simultaneously, the ruptured tumor cells can release their progeny OVs to continue infecting the remaining tumor cells, playing a role in continuously killing tumor cells (Russell et al., 2012; Lawler et al., 2017). After all the tumor cells are infected and cleared by the virus, the virus generally breaks the immune system’s tolerance and triggers an immune response, which results in its elimination (Russell and Barber, 2018). Therefore, as an emerging cancer treatment approach, OVs treatment has significant research prospects and is of great research importance.

With the development of genetic engineering, several OVs treatment has been applied, such as the marketing of Rigvir (Doniņa et al., 2015; Alberts et al., 2018) was approved in Latvia, H101 (Liang, 2018) in China, T-VEC (Wall and Baldwin-Medsker, 2017) in the United States, and the Japanese herpes simplex virus Deltyact (Teserparturev/G47 ∆; Frampton, 2022). However, these viruses have not been particularly widely used after being marketed due to the different structures and biological characteristics of oncolytic viruses. Their production processes and quality control are quite diverse. At present, the understanding of their biological mechanism of action and the quality of oncolytic viruses used for treatment are not comprehensive, which poses great challenges for the research and application of oncolytic viruses in cancer treatment. Moreover, there is currently a lack of comprehensive reviews on clinical trials of most oncolytic viruses in the research field. Therefore, our study aims to fill this gap.

In this review, we will focus on the diversity of OVs, the potential harm to the human body, the high efficiency of killing tumor cells, and their therapeutic methods, and clarify the great role of OVs in the treatment of tumor cells. By searching the PubMed database and ClinicalTrials.gov, we collected many articles and data about OVs in clinical studies and summarized the treatment methods of most OVs and the efficacy of combination therapy.

## 2. Development, species, and research progress of oncolytic viruses

### 2.1. The development of oncolytic viruses

In 1896, a case of leukemia was reported in which the white blood cells of a woman infected with the influenza virus decreased dramatically (Tian et al., 2022). The phenomenon that cancer remission following influenza infection made people realize that viruses and tumors, as diseases that are difficult to cure, may interact with each other. After that, cases of tumor reduction or disappearance following virus infection were reported one after another. In 1912, an Italian doctor discovered that a cervical cancer patient’s tumor began to recede after a rabies vaccination (Das et al., 2021). Since then, scientists conducted clinical trials using various wild-type viruses to treat tumors from the 1950s to the 1980s. However, due to limited medical conditions, technology, and understanding of viruses and tumor mechanisms, viruses did not revolutionize tumor treatment, and the development of oncolytic viruses was slow (Moore, 1952; Southam and Moore, 1952; Asada, 1974). With advancements in modern genetic engineering and research on viral genes, designing and manipulating viruses using genetic engineering has emerged as a promising approach for OVs. In 1991, the first OV was used to inhibit the growth of glioma in mice (Ezzeddine et al., 1991), and the development of OVs began to advance rapidly. In 1996, research proved that the adenovirus with an E1B55K gene mutation could selectively replicate in p53 defective tumor cells (Bischoff et al., 1996), and then the genetically modified adenovirus ONYX-015 entered phase I clinical trials (Rothmann et al., 1998). In 2004, RIGVIR was approved for melanoma treatment in Latvia, followed by H101 in China in 2005, and T-VEC in the United States and Europe in 2015 (Bilsland et al., 2016; Kaufman et al., 2022; Zhang et al., 2022), marking the maturity of oncolytic virus technology and recognition of the efficacy of oncolytic virus. In 2017, Cell reported that the combination of oncolytic virus T-VEC and PD-1 anti-tumor drug Keytruda was used to treat melanoma (Ribas et al., 2017), setting off an upsurge of research on oncolytic virus combined therapy. In 2021, Deltyact (Teserpatricev/G47 ∆) in Japan was approved for listing (Zeng et al., 2021). Until now, numerous genetically modified OVs have undergone various clinical trials and achieved notable results (Macedo et al., 2020), making oncolytic virus one of the promising tumor immunotherapy methods in clinics.

### 2.2. Introduction and development of some oncolytic viruses

Current research shows that the size of oncolytic viruses is mostly distributed between 20 and 200 nm. Selective replication in tumor cells is a critical characteristic of oncolytic viruses for tumor immunotherapy. The replication ability of OV depends on the viral infection ability, the characteristics of tumor cells, and the host resistance (Sostoa et al., 2020). Not all viruses are suitable for use as oncolytic viruses, and their selection depends on factors such as the potential pathogenicity, immunogenicity, and genes encoding the therapeutic ability of the virus (Russell et al., 2012). Several oncolytic viruses are currently in use, including adenovirus (Ad; Gállego Pérez-Larraya et al., 2022; Nisar et al., 2022; Yngve et al., 2022), herpes simplex virus (HSV; Kawamura et al., 2022; Scanlan et al., 2022; Shayan et al., 2022), vaccinia virus (VV; Zhou et al., 2020; Yang Z. et al., 2021), reovirus (Seyed-Khorrami et al., 2021; Yang C. et al., 2021), poliovirus (Ghajar-Rahimi et al., 2022; Wei et al., 2022), coxsackie virus (CV; Lu et al., 2020; Sarwar et al., 2021), Newcastle disease virus (NDV; Chan et al., 2021), vesicular stomatitis virus (VSV; Sarwar et al., 2021), myxoma virus (Villa et al., 2022), and some enteroviruses (Xu et al., 2021; Kolyasnikova et al., 2023).

Nonetheless, the ability to selectively infect certain tumors may also limit the virus’s applicability to specific tumor types. Su et al. found a way to overcome this limitation by employing the survivin promoter as a regulatory element to direct the adenovirus toward cancer cells (Su et al., 2022). Additionally, they reduced the expression of Elb-55kD to augment the virus’s tumor selectivity, allowing for adenovirus replication in various tumor cells while minimizing excessive viral replication in normal cells. Consequently, the modified adenovirus demonstrated a broad-spectrum anti-cancer effect.

#### 2.2.1. Adenovirus

Adenovirus (Ad) is a type of double-stranded DNA virus. Ads are the primary focus of oncolytic virus research. Oncolytic adenovirus can infect almost all types of cells and is easy to prepare and purify. However, it also has the disadvantage that Ad in the body is easy to cause induction of autoimmunity to reject the Ad. Back in 2005, China approved the first OV, an Ad called H101 (Liang, 2012; Wei et al., 2018), for the treatment of squamous cell carcinoma of the head and neck (SCCNH; Garber, 2006). Ads are used to treat several types of cancer, including melanoma (García et al., 2019; Milenova et al., 2021), lung cancer (Watanabe et al., 2006), colorectal cancer (Kuhn et al., 2008), prostate adenocarcinoma (PRAD ; Chen et al., 2001; DeWeese et al., 2001; Ramesh et al., 2006; Small et al., 2006; Huang et al., 2008; Burke et al., 2012; Rojas-Martínez et al., 2013; Packiam et al., 2018; Oronsky et al., 2022), malignant glioma (MG; Chiocca et al., 2004; Lang et al., 2018; Kieran et al., 2019; Carpenter et al., 2021), ovarian cancer (OC; Kimball et al., 2010; Kim et al., 2013; Vassilev et al., 2015), head and neck cancer (HNC; Chang et al., 2009; Nakajima et al., 2009), and solid tumors (Lin et al., 2007; Li et al., 2009; Nemunaitis et al., 2010; Nokisalmi et al., 2010; Pesonen et al., 2012; Machiels et al., 2019).

Adenovirus is commonly modified by four methods: the first method involves modifying the gene sequence in the E1 region. For example, the E1b55K of the virus does not prevent the apoptosis pathway in normal cells, but it does in tumor cells (Cheng et al., 2015). The second method involves using tumor-specific promoters to replace the necessary promoters in Ad, such as the promoters of telomerase reverse transcriptase, to regulate E1A gene expression in cancer cells with high levels of telomerase reverse transcriptase (Tazawa et al., 2020). The third method is to modify the Ad capsid protein to make the virus bind more selectively to tumor cells (Wang et al., 2016; Franke et al., 2023). Furthermore, inserting genes to express pro-apoptotic proteins can enhance the ability of the virus to kill tumor cells (Cerullo et al., 2010). Some of the oncolytic adenoviruses under study are listed in Table 1.

**Table 1:** Some oncolytic adenoviruses are under study.

Number	The virus name	Modification	Phase	Combination	Applicable disease	Reference
1	H101	Deletion in the E1B-55 k gene and four deletions in the E3 gene	Listed	Chemotherapy	EC, NPC, and HNC	Xu et al. (2021); Garber (2006); Liang (2012); Wei et al. (2018); Jin et al. (2021)
2	ADV-TK	Deletion of the ICP34.5 gene and ICP47 gene	Phase III	Chemotherapy, radiotherapy, and PD-1	HCC, lung cancer, MG, PAAD, PRAD, and SCCHN	Rojas-Martínez et al. (2013); Kieran et al. (2019)
3	CG0070	Carriage of the cancer-selective promoter E2F-1	Phase III	/	BDC	Ramesh et al. (2006); Burke et al. (2012); Packiam et al. (2018); Oronsky et al. (2022)
4	E10A	Deletion in the E1 and partial E3 region	Phase III	Cisplatin, paclitaxel	Solid tumor	Lin et al. (2007); Li et al. (2008)
5	DNX-2401 (CCTG-602, Delta-24-RGD)	E1A 24 bp-deleted	Phase II	PD-1	Glioblastoma	Kimball et al. (2010); Pesonen et al. (2012); Kim et al. (2013); Hemminki et al. (2015); Lang et al. (2018); Carpenter et al. (2021); Oronsky et al. (2022)
6	KH901	The sequence of the promoter was modified to include two E2F-1 binding sites	Phase II	Chemotherapy	SCCHN	Shen et al. (2007a,b); Chang et al. (2009); Wei et al. (2018)
7	LOAd703	Deletion of 24 base pairs within the E1A region’s retinoblastoma binding domain	Phase II	PD-1, paclitaxel	PAAD, MM	Eriksson et al. (2017); Wenthe et al. (2020); Wenthe et al. (2021); Oronsky et al. (2022)
8	OBP-301	hTERT promoter sequence inserted into the DNA sequence before the E1 genes	Phase II	Chemotherapy	Solid tumor	Watanabe et al. (2006); Huang et al. (2008); Nakajima et al. (2009); Nemunaitis et al. (2010); Huang et al. (2008); Shirakawa et al. (2021); Oronsky et al. (2022)
9	ONCOS-102 (CGTG-102, Ad5/3-D24-GMCSF)	Deletion of 24 base pairs in the retinoblastoma binding domain of the E1A region	Phase II	Chemotherapy, PD-1	CC, melanoma, MPM, OC, and PRAD	Kanerva et al. (2013); Bramante et al. (2014); Siurala et al. (2015); Vassilev et al. (2015); Oronsky et al. (2022)
10	ONYX-015 (dl1520, CG7870)	Deletion of 827 bp in the E1B region	Phase II	Chemotherapy, radiotherapy	BC, PRAD, and SCCHN	Heise et al. (1997); Kirn et al. (1998); Reid et al. (2002); Chiocca et al. (2004); Galanis et al. (2010); Dilley et al. (2005); Small et al. (2006); Parmar et al. (2022)
11	ORCA-010	T1 mutation in the E3/19K gene, 24 mutations in the E1A region	Phase II	/	PRAD	Dong et al. (2014); Milenova et al. (2021)
12	ColoAd1 (Enadenotucirev)	Generated through directed evolution of the adenovirus	Phase I	Radiotherapy	BDC, CC, HCC, OC, and renal carcinoma	Kuhn et al. (2008); Garcia-Carbonero et al. (2017); Machiels et al. (2019)
13	CV706	Insertion of a minimal promoter-enhancer construct by human PSA gene (PSE) 5′ of E1A and 3′ of the E1A promoter	Phase I	Chemotherapy, radiotherapy	PRAD	Chen et al. (2001); DeWeese et al. (2001)
14	H103	Carriage of the heat shock protein (HSP) 70 gene	Phase I	/	Solid tumor	Li et al. (2009); Wei et al. (2018)
15	ICOVIR5	Deletion of the E1A region responsible for retinoblastoma protein binding, an RGD sequence inserted at the fiber knob	Phase I	/	Melanoma, retinoblastoma	Abate-Daga et al. (2011); Rincon et al. (2017); García et al. (2019)
16	ICOVIR-7	Modification of the E2F promoter and an Rb-binding site deletion of E1A	Phase I	/	Solid tumor	Rojas et al. (2009); Nokisalmi et al. (2010)
17	TILT-123	E2F promoter and the 24-base-pair deletion in constant region 2 of E1A	Phase I	PD-1	Solid tumor	Havunen et al. (2021)
18	VCN-01	E1A 24 bp-deleted, partially E3 deleted	Phase I	Paclitaxel, gemcitabine	Solid tumor	Rodriguez-Garcia et al. (2015); Pascual-Pasto et al. (2019)

BDC, bladder cancer; CC, colorectal cancer; EC, esophageal carcinoma; GC, gastrointestinal cancer; HCC, hepatocellular carcinoma; HNC, head and neck cancer; MG, malignant glioma; MM, multiple myeloma; MPM, malignant pleural mesothelioma; NPC, nasopharyngeal carcinoma; OC, ovarian cancer; PAAD, pancreatic adenocarcinoma; PD-1, programmed cell death protein 1; PRAD, prostate adenocarcinoma; and SCCNH, squamous cell carcinoma of the head and neck.

#### 2.2.2. Herpes simplex virus

Herpes virus is a group of DNA viruses enclosed by a cellular membrane. It exhibits strong replication ability and is commonly used in genetic engineering. Herpesvirus has numerous advantages, including a wide range of infected hosts, high infection efficiency, large genome capacity, clear gene and protein functions, and low pathogenicity. Research on HSV-1 in the herpesvirus group has shown that it has a positive effect on the treatment of tumor cells. HSV has demonstrated a significant effect on melanoma (Senzer et al., 2009; Kaufman et al., 2010; Andtbacka et al., 2015; Johnson et al., 2015; Killock, 2015; Puzanov et al., 2016; Chesney et al., 2018a,b; Andtbacka et al., 2019; Yun et al., 2022), breast cancer (BC; Teshigahara et al., 2004; Gholami et al., 2014; Fan et al., 2021; Parmar et al., 2022), HNC (Mace et al., 2008; Harrington et al., 2010), glioblastoma (Papanastassiou et al., 2002; Kanai et al., 2011; Patel et al., 2016; Omar et al., 2021), PRAD (Wang et al., 2019), peritoneal cancer (Nakano et al., 2005), colorectal cancer (CC; Kemeny et al., 2006; Geevarghese et al., 2010), pancreatic cancer (PAAD; Nakao et al., 2011; Kelly et al., 2016; Hirooka et al., 2018), solid tumor (Zhang et al., 2021), and other tumor cells. Currently, there are several strategies for HSV virus transformation, including deleting UL39 and other genes required for replication in nondividing cells and deleting the neural virulence gene ICP34.5 and immune escape-related gene ICP47 (Campadelli-Fiume et al., 2011). For example, in 2015, the T-VEC virus became the first OV approved by the FDA for the treatment of malignant melanoma (Johnson et al., 2015). The T-VEC deleted ICP34.5 and ICP47 genes and inserted foreign genes that can express immune-activating protein granulocyte-macrophage colony-stimulating factor (Harrington et al., 2015). The protein encoded by the ICP34.5 gene is critical for the infection of healthy cells. Therefore, deleting the ICP34.5 gene is a strategy to improve the virus’s tumor selectivity (Russell and Barber, 2018; Jin et al., 2021; Tian et al., 2022). However, deleting the ICP34.5 gene may make the virus more susceptible to clearance by immune cells (Yin et al., 2017). To avoid this, researchers have deleted the ICP47 gene to prevent the virus from being cleared by immune cells (Jin et al., 2021). And Japan’s Deltyact (testerpaturev/G47 Δ) also deleted these two genes and mutated the ICP6 gene (Frampton, 2022). The second strategy involves improving the targeting of the virus to tumor cells. For example, the glycoprotein of the virus can be modified to enhance the targeting of the virus to tumor cells (Menotti et al., 2008). In addition, the specific recognition receptor can also be constructed into the gene sequence of the virus to build a virus mutant that recognizes and expresses tumor cell-related receptors (Menotti et al., 2006; Grandi et al., 2010). The third strategy involves inserting immune stimulator-related genes to enhance local immune toxicity, such as GM-CSF, PD1/PD-L1, IL-2, etc. Table 2 shows some of the HSVs currently under study.

**Table 2:** Some herpes simplex viruses are under study.

Number	The virus name	Modification	Phase	Combination	Applicable disease	Reference
1	T-VEC	Deletion of the ICP34.5 gene and ICP47 gene	Listed	PD-1, CTLA-4	Melanoma	Andtbacka et al. (2015); Killock (2015); Johnson et al. (2015); Puzanov et al. (2016); Ribas et al. (2017); Chesney et al. (2018a, 2018b); Andtbacka et al. (2019) Jin et al. (2021); Chaurasiya et al. (2021)
2	G47Delta	Deletion in the gene ICP47	Phase III	Temozolomide, chemotherapy, and radiotherapy	BC, gastric cancer, melanoma, and PRAD	Wang et al. (2019); Fan et al. (2021); Chaurasiya et al. (2021)
3	G207	Deletion of the ICP34.5 gene and insertion of the *Escherichia coli* lacZ gene into ICP6	Phase II	/	Glioblastoma, peritoneal cancer	Nakano et al. (2005); Markert et al. (2014); Chaurasiya et al. (2021)
4	HSV1716	Deletion of the ICP34.5 gene	Phase II	chemotherapy	glioblastoma, HCC, melanoma, MPM, and SCCHN	Papanastassiou et al. (2002); Mace et al. (2008); Streby et al. (2017); Streby et al. (2019); Chaurasiya et al. (2021)
5	NV1020	Deletion of α0, α4, γ34.5, UL56, and UL24 genes	Phase II	Chemotherapy	CC, HCC	Kemeny et al. (2006); Geevarghese et al. (2010); Yun et al. (2022)
6	OrienX010 (GM-CSF)	Deletions in ICP34.5 and ICP47	Phase II	PD-1, Dacarbazine	HCC, lung cancer, melanoma, and PAAD	Senzer et al. (2009); Kaufman et al. (2010); Harrington et al. (2010); Yun et al. (2022); Wei et al. (2018)
7	HF10	Absence of UL43, UL49.5, UL55, and UL56	Phase II	Chemotherapy, paclitaxel, and gemcitabine	Solid tumor	Teshigahara et al. (2004); Nakao et al. (2011); Hirooka et al. (2018); Jin et al. (2021)
8	M032	Substitution of ORF and P γ134.5 gene with α27-tk	Phase I	/	Glioblastoma	Patel et al. (2016); Omar et al. (2021); Yun et al. (2022)
9	NV1066	Deletions of UL23	Phase I	/	BC, PAAD	Gholami et al. (2014); Kelly et al. (2016)
10	ONCR-177	Insertion of the miRNA sequence into ICP4, ICP27, and UL8	Phase I	PD-1	Solid tumor	Haines et al. (2021); Chaurasiya et al. (2021)
11	VG161	Deletion of the γ34.5	Phase I	CAR-T	Solid tumor	Chouljenko et al. (2020); Yun et al. (2022)

BC, breast cancer; CAR-T, chimeric antigen receptor T-cell immunotherapy; CC, colorectal cancer; CTLA-4, cytotoxic T-lymphocyte-associated protein 4; HCC, hepatocellular carcinoma; MPM, malignant pleural mesothelioma; PAAD, pancreatic adenocarcinoma; PD-1, programmed cell death protein 1; and SCCNH, squamous cell carcinoma of the head and neck.

#### 2.2.3. Vaccinia virus

Vaccinia virus is a giant double-stranded DNA virus that comes in two forms. It is a vaccine against smallpox infection with detailed human safety data. And have multiple mechanisms of action that target human and rodent tumors, making the virus more effective. For example, the Pexa-VEC contains a deletion of viral thymidine kinase gene (Chaurasiya et al., 2021). The advantage of utilizing VV to treat tumors is that it replicates quickly. The genome does not integrate itself into the host cell, and the genome is large enough to insert whole segments of foreign genes. VV is effective in targeting hepatocellular carcinoma (HCC) (Park et al., 2008; Heo et al., 2013), melanoma (Hwang et al., 2011), CC (Park et al., 2015), HNC (Mell et al., 2017), peritoneal cancer (Lauer et al., 2018), OC (Mori et al., 2019), BC (Beguin et al., 2020), glioblastoma (Breton et al., 2010), and some other tumors (Cripe et al., 2015; Downs-Canner et al., 2016; Peng et al., 2018). At present, the first modification strategy for the vaccinia virus is to delete the genes necessary for virus replication in normal cells, such as thymidine kinase (Parato et al., 2012), and vaccinia growth factor (McCart et al., 2001), so that the virus can only replicate in tumor cells. The second strategy is to insert genes encoding specific tumor-associated antigens or tumor-specific antigens to increase specific T-cell-mediated immune responses. In addition, immunogenicity is further enhanced by arming the virus or modifying the viral vector with immune-stimulating molecules such as interleukins and colony-stimulating factors (Guo et al., 2019). Some of the VV under-studying can be seen in Table 3.

**Table 3:** Some vaccinia viruses are under study.

Number	The virus name	Modification	Phase	Combination	Applicable disease	Reference (PMID)
1	Pexa-VEC (JX-594)	Deletion of viral thymidine kinase gene	Phase III	Sorafenib	BC, CC, HCC, melanoma, and renal carcinoma	Park et al. (2008); Hwang et al. (2011); Breitbach et al. (2011a); Heo et al. (2013); Cripe et al. (2015); Park et al. (2015); Chaurasiya et al. (2021)
2	GLV-1 h68 (GL-ONC1)	The F14.5 L, J2R and A56R genes are replaced with Ruc-GFP, β-glucuronidase, and β-galactosidas	Phase II	Chemotherapy, radiation therapy	Solid tumor	Mell et al. (2017); Lauer et al. (2018); Mori et al. (2019); Chaurasiya et al. (2021)
3	T601 (TG6002)	Deletion of the thymidine kinase gene	Phase II	5-Fluorouracil	CC, GC, HCC	Beguin et al. (2020); Beguin et al. (2021); Jin et al. (2021)
4	MVA-FCU1	Containing the yeast-originated transgene FCU1	Phase I	5-Fluorouracil	HCC, glioblastoma	Breton et al. (2010); Husseini et al. (2017)
6	vvDD	Deletion of J2R and C11R genes	Phase I	/	BC, CC, HCC, melanoma, PAAD, SCCNH	Downs-Canner et al. (2016); Chaurasiya et al. (2021)

BC, breast cancer; CC, colorectal cancer; GC, gastrointestinal cancer; HCC, hepatocellular carcinoma; PAAD, pancreatic adenocarcinoma; and SCCNH, squamous cell carcinoma of the head and neck.

#### 2.2.4. Newcastle disease virus

Newcastle disease virus is an RNA virus that causes Newcastle disease in birds but has mild symptoms in humans. It is a typical example of oncolytic viruses. Recent studies have shown that NDV combined with radiotherapy and combined point inhibitors has been shown to improve the clearance rate of melanoma in mice (Vijayakumar et al., 2019). Similarly, NDV infects melanoma (Lazar et al., 2010), HNC (Prince et al., 2005), glioma (Freeman et al., 2006), solid tumor (Pecora et al., 2002), and other tumors (Yaacov et al., 2008). NDV can increase its number in tumor cells by using the B cell lymphoma overexpressed by tumor cells (Mansour et al., 2011). NDV is mainly used for immune regulation, such as by introducing IL-2 (Zamarin et al., 2009) or reverse genetics (Vigil et al., 2007) to improve the efficacy of tumor lysis. One example shows that the L289A mutation of the F gene has increased cytotoxicity, which can be used to treat HCC patients with improved safety and effectively (Altomonte et al., 2010). Some of the NDVs under-studying can be seen in Table 4.

**Table 4:** Newcastle disease virus under study.

Number	The virus name	Modification	Phase	Combination	Applicable disease	Reference
1	NDV-HUJ	Passaged multiple times in specific eggs	Phase I	/	Glioblastoma	Freeman et al. (2006); Yaacov et al. (2008); Lazar et al. (2010)
2	PV701	Naturally occurring virus with oncolytic features	Phase I	/	Solid tumor	Pecora et al. (2002); Prince et al. (2005); Laurie et al. (2006); Lorence et al. (2007)

#### 2.2.5. Reovirus

A naturally occurring double-stranded RNA virus, reovirus normally infects the respiratory and intestinal systems of mammals. The oncolytic ability of reovirus is associated with the good replication and dissolution of these cells found in various cancer cell lines. The virus works against a variety of cancers, including BC, SCCNH, melanoma (Galanis et al., 2012; Mahalingam et al., 2017), multiple myeloma, PAAD, CC (Goel et al., 2020; Chaurasiya et al., 2021), glioma (Forsyth et al., 2008; Kicielinski et al., 2014), and lung cancer. Reovirus likes to replicate in cells with dysregulated growth factor signaling cascade (Shmulevitz et al., 2005). In tumor cells lacking anti-virus PKR signal transduction defects, tumor lysis can occur directly. At the same time, cytotoxicity mediated by NK cells and T cells is a determining factor for anti-tumor efficacy (Rajani et al., 2016). Transformed cells that activate the Ras signaling pathway are tolerant to reovirus by enhancing viral membrane detachment, increasing particle infectivity, and viral apoptosis release (Marcato et al., 2007). The reovirus situation can be seen in Table 5.

**Table 5:** Reovirus under study.

Number	The virus name	Modification	Phase	Combination	Applicable disease	Reference
1	REOLYSIN® (Pelareorep)	Naturally occurring virus with oncolytic features	Phase III	Chemotherapy, Paclitaxel, PD-1	BC, CC, FTC, melanoma, MG, OC, PAAD, and PRAD	Forsyth et al. (2008); Comins et al. (2010); Adair et al. (2012); Galanis et al. (2012); Morris et al. (2013); Chakrabarty et al. (2013); Kicielinski et al. (2014); Mahalingam et al. (2017); Goel et al. (2020); Chaurasiya et al. (2021)
2	RT3D	Naturally occurring virus in the human body with oncolytic features	Phase I	Chemotherapy, radiotherapy	HNC	White et al. (2008); Karapanagiotou et al. (2012); Roulstone et al. (2015)

BC, breast cancer; CC, colorectal cancer; FTC, fallopian tube carcinoma; HNC, head and neck cancer; MG, malignant glioma; OC, ovarian cancer; PAAD, pancreatic adenocarcinoma; PD-1, programmed cell death protein 1; and PRAD, prostate adenocarcinoma.

#### 2.2.6. Some other viruses

In 2004, Latvia launched Rigvir (Doniņa et al., 2015), a naturally occurring virus with oncolytic features, which is used to treat melanoma, CC, PAAD, BDC, renal carcinoma, PRAD, lung cancer, uterine cancer, lymphoid sarcoma, gastrointestinal cancer (GC), and other cancers (Alberts et al., 2016; Brokane et al., 2019). A case study involving the use of Rigvir therapy demonstrated the therapeutic value of this treatment in advanced melanoma. The patient, a 1972-born female, was diagnosed with malignant melanoma in the lumbar region. Following surgical and chemotherapy interventions, the tumor showed a poor response and was associated with severe systemic side effects (Alberts et al., 2016). In February 2013, the patient began receiving Rigvir treatment as the sole therapy. After treatment, the size of the inguinal lymph nodes was reduced by half, and the patient’s condition remained stable until the time of publication in 2016. These findings highlight the significant therapeutic potential of Rigvir in late-stage melanoma (Alberts et al., 2016).

Coxsackie virus (CV) is an enterovirus that infects people with symptoms such as fever, cough, and cold. Research by British scientists has shown that CV has the potential to target, infect, and destroy cancer cells in patients with BDC (Annels et al., 2019). CV is also used against many types of cancer (Bradley et al., 2014; Li et al., 2022), including melanoma, BDC, lung cancer, and PRAD. It can enter tumor cells through cells *via* intercellular adhesion molecule 1 (CD54), decal accelerating factor (CD55), or connectors that may be overexpressed in multiple myeloma, melanoma, breast cancer, and other tumor cells (Shafren et al., 2004; Au et al., 2007; Guo et al., 2014).

Poliovirus is a tiny RNA enterovirus that invades nerve cells, and humans are the only host for the virus. Poliovirus can be used as a new cancer treatment tool (Brown et al., 2014). The poliovirus solely relies on the CD155 receptor to enter the host cell and CD155 is abundant in malignant cells (Gromeier et al., 2000). This CD155 receptor may damage the response of anti-tumor immune cells and be overexpressed by tumor cells (Carlsten et al., 2009). Poliovirus is used to treat glioblastoma, BDC, melanoma, and other symptoms. In 2018, PVSRIPO, a treatment developed at Duke University, significantly improved long-term survival in patients with recurrent glioma. However, some studies have shown the cytotoxicity of poliovirus (Goetz et al., 2010; Brown and Gromeier, 2015).

In 2014, the research group Yan found that the natural oncolytic virus M1 can selectively infect a variety of human cancers with ZAP deficiency (Lin et al., 2014), including Hepatocellular carcinoma (HCC), BDC, and melanoma, and it has no toxic side effects on normal cells (Zhang et al., 2016; Cai and Yan, 2021).

There are also relevant studies on other oncolytic viruses, such as measles virus (Hasegawa et al., 2006; Galanis et al., 2010; Zhang et al., 2012; Aref et al., 2016; Dispenzieri et al., 2017; Packiriswamy et al., 2020), H1 Parvovirus (Geletneky et al., 2017; Hajda et al., 2017), myxoma virus (Cornejo et al., 2020), Seneca Valley virus (Rudin et al., 2011; Burke et al., 2015; Burke, 2016; Schenk et al., 2020), and VSV (Tober et al., 2014; Schreiber et al., 2019), to prove the development of these OVs in fighting tumors.

Table 6 shows some of the viruses currently under study.

**Table 6:** Some other oncolytic viruses are under study.

Number	Virus type	Modification	The virus name	Phase	Applicable disease	Reference
1	EHCO-7	Naturally occurring virus with oncolytic features	Rigvir	Listed	BCD, CC, melanoma, PAAD, PRAD, renal carcinoma, and SCLC	Doniņa et al. (2015); Alberts et al. (2016); Brokane et al. (2019)
2	Coxsackie virus	Naturally occurring virus in the human body with oncolytic features and without pathogenicity	CVA21 (Cavatak)	Phase II	BDC, melanoma, SCLC, and PRAD	Bradley et al. (2014); Annels et al. (2019); Li et al. (2022)
3	Parainfluenza virus	Naturally occurring virus after removal of pathogenicity	HVJ (SeV)	Phase II	PRAD	Kaneda et al. (2010); Kiyohara et al. (2020); Fujita et al. (2020)
4	Influenza A virus	Naturally occurring virus with oncolytic features	M1	Phase II	BCD, CC, HCC, melanoma, NPC, and PRAD	Zhang et al. (2016); Cai and Yan (2021)
5	H-1PV	Naturally occurring virus with oncolytic features	ParvOryx	Phase II	Glioblastoma	Hajda et al. (2017); Geletneky et al. (2017)
6	Measles virus	Insertion of the human CEA gene upstream of the MV N gene	MV-CEA	Phase I	Glioblastoma, HCC, OC	Hasegawa et al. (2006); Galanis et al. (2010); Zhang et al. (2012)
7	SVV	Naturally occurring virus with oncolytic features	NTX-010 (SVV-001)	Phase I	Carcinoid, NEC	Rudin et al. (2011); Burke et al. (2015); Burke (2016); Schenk et al. (2020)
8	Poliovirus	Exchanging of cognate internal ribosomal entry site with human rhinovirus type 2	PVSRIPO	Phase I	BC, MG, melanoma, and glioblastoma	Goetz et al. (2010); Brown et al. (2014); Brown and Gromeier (2015)
9	VSV	Glycoprotein replaced by the lymphocytic choriomeningitis virus	VSV-GP	Phase I	Glioblastoma, HCC, and melanoma	Tober et al. (2014); Schreiber et al. (2019)

BDC, bladder cancer; CC, colorectal cancer; GC, gastrointestinal cancer; H-1PV, H1 Parvovirus; HCC, hepatocellular carcinoma; NEC, neuroendocrine carcinoma; NPC, nasopharyngeal carcinoma; OC, ovarian cancer; PAAD, pancreatic adenocarcinoma; PRAD, prostate adenocarcinoma; SCCNH, squamous cell carcinoma of the head and neck; and SVV: seneca valley virus.

### 2.3. Clinical research status of oncolytic virus

By visiting Clinicaltrials.gov and searching for the keyword “oncolytic,” 186 clinical study registries were identified, 147 of which had detailed clinical data. The remaining clinical trial registrations were rejected for further analysis because their status was withdrawn, terminated, unknown, or suspended and because the articles did not conform to the contents of this review. Further analysis of 147 clinical study registries was performed, and multiple variables were assessed, including clinical trial phase, number of patients, route of administration, and indications of application. A complete list of clinical study registries is in Tables 1-6.

#### 2.3.1. Research stage

**Figure 1:**
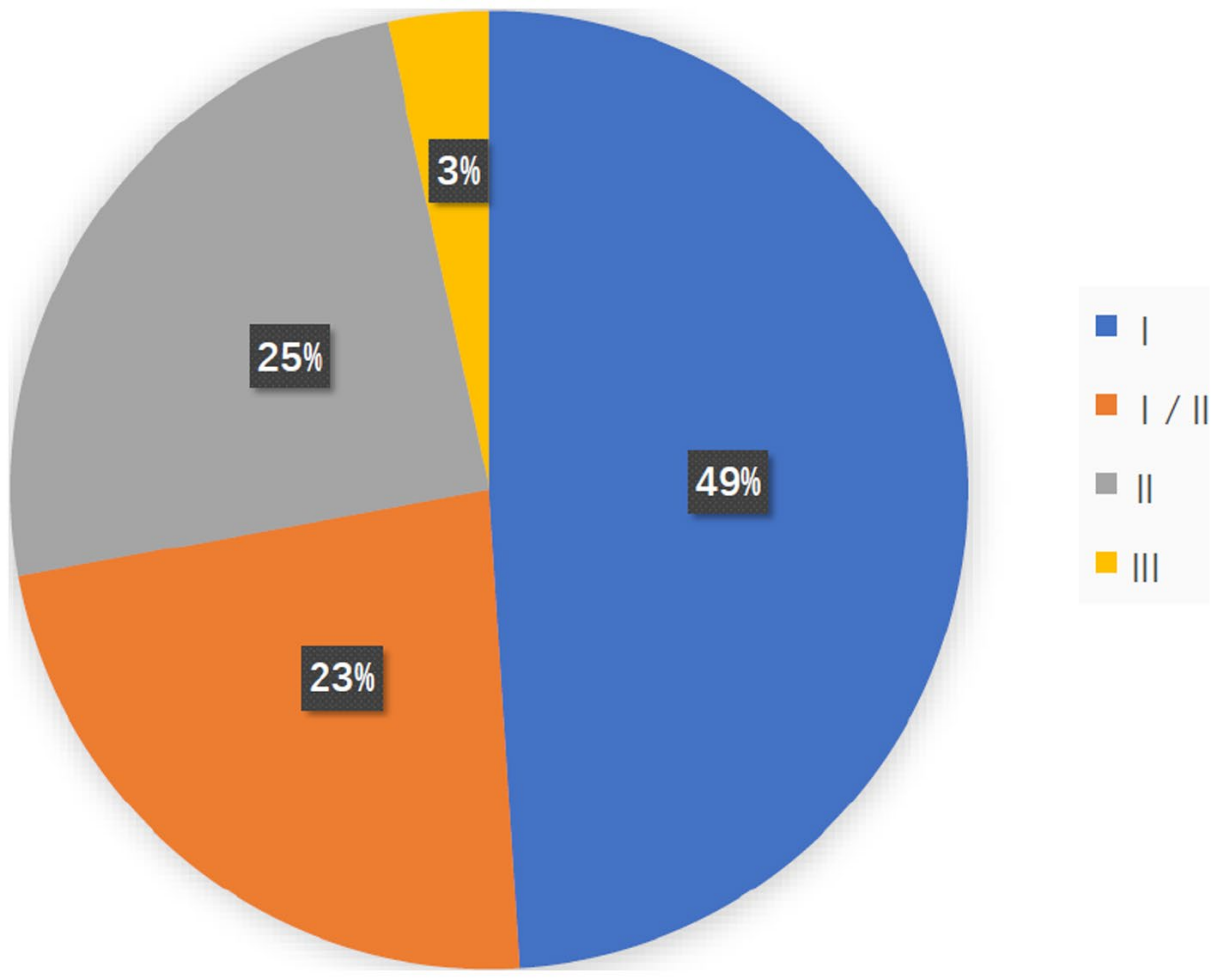
The pie chart shows the study stage of 147 clinical studies, most of which were in Phase I (*n* = 72, 48.9%), Phase I/II (*n* = 34, 23.1%), and Phase II (*N* = 36, 24.5%) and Phase III (*n* = 5, 3.40%).

A total of 7,776 cancer patients were enrolled and completed 147 clinical trials. Most clinical studies were in Phase I (*n* = 72), Phase I/II (*n* = 34), Phase II (*n* = 36), and Phase III (*n* = 5; Figure 1). This shows that OVs research is currently in its early stages and has great potential for development.

#### 2.3.2. Route of administration and indications of application

**Figure 2:**
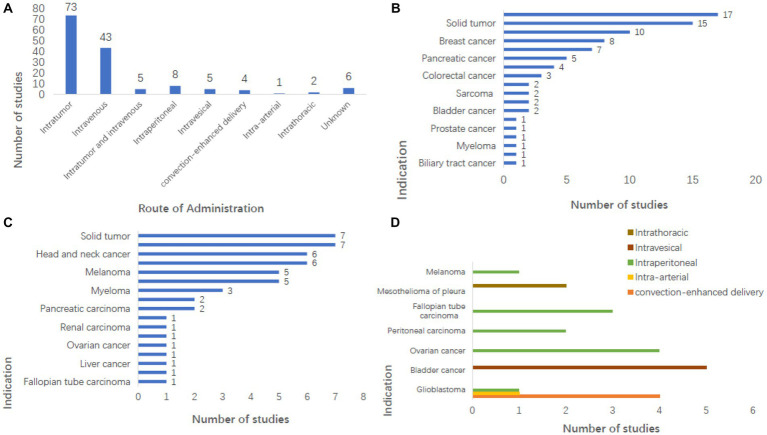
Analysis of administration pattern and indications in clinical study registry. **(A)** Intratumoral injection (*n* = 73) and intravenous injections (*n* = 43) were the main administrative methods in the study, and the number of other administrative methods is shown in the figure. **(B)** Shows the number of studies on tumors where intratumoral injection can be used, among which melanoma has the largest number of studies (*n* = 17, 20.5%), which are mostly used in benign solid tumors but also some malignant tumors. **(C)** Shows the number of studies of tumors that can be injected intravenously, the majority of which are malignant solid tumors. **(D)** Showing tumors that can be treated by administration other than intratumoral and intravenous injections, it can be seen that glioblastoma is a tumor that can be treated by virtually every administration method.

A collation of clinical study registries found that 73 trials (49.7%) involved intratumor injection, and 43 (29.3%) involved intravenous injection. There were 5 (3.4%) experiments of both intratumor injection and intravenous injection, eight involving Intraperitoneal administration, five involving intravesical injections, four involving Convection-enhanced delivery (CED), two involving intraperitoneal injections, one involving intra-arterial injection, and six without explanation of how to conduct the administration (Figure 2A).

Tumor site administration is also referred to as an intratumoral injection. Currently, intratumoral virus injection, which allows the virus to expand in tumor cells, is the preferred method for most OVs in clinical and development stages. However, intratumoral injection becomes very difficult once tumor cells develop total metastasis due to the need to accurately locate tumor cells and the difficulty of monitoring and controlling the therapeutic effect. Although OVs will not migrate to the location of the metastatic tumor, they will activate the host’s anti-tumor response to control the metastatic tumor (Twumasi-Boateng et al., 2018; Hinterberger et al., 2021). The intratumoral injection is used for melanoma, glioblastoma, BC, liver cancer, PAAD, HNC, CC, BDC, and other benign solid tumors (Figure 2B). A total of 4,411 patients received intratumoral injections, including over 1,300 in the melanoma study. At the same time, many articles have studied the effect of intratumoral injection (Li et al., 2008; Nakao et al., 2011; Morris et al., 2013; Streby et al., 2017; Hirooka et al., 2018).

Intravenous injections avoid the need to look for specific tumor cells and allow for a single treatment option for the patient, something that intratumoral drugs cannot. Intravenous injection can accurately deliver the drug to a location that the local injection cannot reach. However, drugs given intravenously need to be minimal or harmless to normal cells in the body, and some neutralizing substances in the blood may neutralize the virus, and the immune system’s response may eliminate the virus too quickly to be effective in treating tumor cells. In addition, the combination of the virus with certain components in the blood limits the delivery route of the virus and makes treatment less effective (Kaufman and Bommareddy, 2019). It may even produce adverse reactions of varying degrees (Imperiale, 2008). And Intravenous injection is one of the main research directions at present (Laurie et al., 2006; Lorence et al., 2007; White et al., 2008; Comins et al., 2010; Breitbach et al., 2011a; Chakrabarty et al., 2013; Park et al., 2015; Garcia-Carbonero et al., 2017; Mell et al., 2017; Streby et al., 2019; Beguin et al., 2021).

Several oncolytic viruses are being tested intravenously, including REOLYSIN®(Pelareorep; Adair et al., 2012; Galanis et al., 2012), Enadenotucirev (ColoAdl; Garcia-Carbonero et al., 2017; Marino et al., 2017; Machiels et al., 2019), HSV1716 (Streby et al., 2019), JX594 (Breitbach et al., 2011b), MV-NIS (Dispenzieri et al., 2017), NDV-HUJ (Freeman et al., 2006), PV701 (Hotte et al., 2007), RT3D (White et al., 2008; Karapanagiotou et al., 2012), vvDD (Downs-Canner et al., 2016), and TG6002 (Beguin et al., 2021). Other OVs such as Seneca Valley virus and Coxsackievirus A21, have also been studied to show the feasibility and safety of intravenous administration (Kennedy et al., 2022). Intravenous injection can be used for lung cancer, HNC, CC, gynecological tumor, and other malignant tumors (Figure 2C). The number of patients who received intravenous injections was 2,198, accounting for 28% of the total.

Intra-arterial injection, in which the virus is selectively transported throughout the body, and because of the limited volume of the blood and target organs, can avoid the reaction of the antibodies in intravenous injection with the virus, which can reduce the effectiveness of the virus. Experiments on the treatment of OVs with intrahepatic artery administration have been conducted (Reid et al., 2002; Fong et al., 2009). Quantum-enhanced delivery can be used for the treatment of glioblastoma. Intravesical injection, intraperitoneal injection, and intrathoracic injection are all used for tumors in the bladder, abdominal cavity, and thoracic cavity (Figure 2D).

Although the effect of intratumoral injection of OVs is good, intravenous and arterial administration also has a certain effect. The elimination of OVs by antibodies in the body, the destruction of viruses by complements, and the clearance of viruses by organs and tissues such as the liver, spleen, and kidney will affect the effect of OVs in treating tumors (Kolb et al., 2015; Roy et al., 2020). Therefore, many studies have attempted to use carrier tools to improve the problem of how to avoid being attacked by the immune system after intravenous administration, such as using liposomes (Iscaro et al., 2022), nanoparticles (Fusciello et al., 2019), immunoliposomes (Li et al., 2016), mesenchymal stem cells (Naji et al., 2019; Franco-Luzon et al., 2020), and other carrier tools.

Mesenchymal stem cells have great advantages as the carrier tool of OVs in systematic administration because they can be isolated from a variety of tissues, have low immunogenicity, and possess chemotaxis to solid tumors (Volarevic et al., 2018). Mesenchymal stem cells can be used as the cell carrier of OVs for multiple injections and as the replication site of OVs to generate more progeny viruses and promote the tumor-killing effect of OVs. In the latest research, mesenchymal stem cells have the characteristics of chemotaxis to solid tumors (Najar et al., 2016; Karagiannis et al., 2017; Vangala et al., 2019; Kostadinova and Mourdjeva, 2020). At the same time, some studies have shown that cytokines secreted by tumor cells are one of the key reasons for inducing mesenchymal stem cells to chemotaxis to tumor cells (Lejmi et al., 2015; Pavon et al., 2018), and which may promote the occurrence of anti-tumor immunity (Morales-Molina et al., 2018).

Although mesenchymal stem cells carrying oncolytic viruses have shown potential efficacy in preclinical studies, but their effectiveness in clinical trials is still being evaluated. They can also promote the occurrence of tumor cells, inhibit apoptosis of tumor cells, and promote tumor metastasis through a variety of mechanisms (Naji et al., 2019; Atiya et al., 2020; Kostadinova and Mourdjeva, 2020). There are some controversies about the delivery of oncolytic virus by mesenchymal stem cells, but in general, the destructive effect on tumors may be greater than the promoting effect.

### 2.4. Combination therapy with oncolytic virus

**Figure 3:**
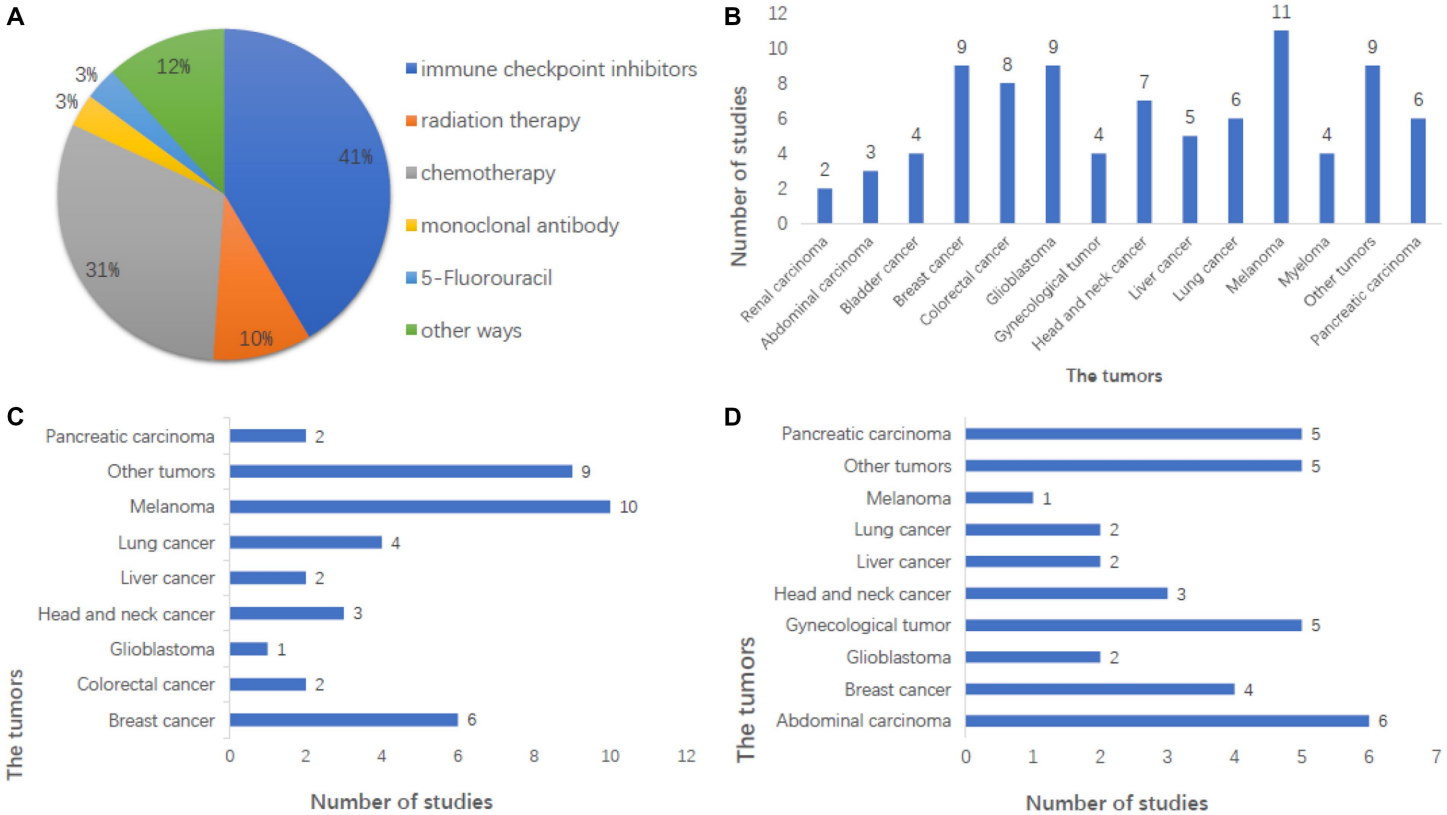
Analysis of combination therapy in the registry of clinical studies. **(A)** Combination therapy in the study was mainly immune checkpoint inhibitors and chemotherapy, and the other combination therapy methods were also studied in A certain number of studies. **(B)** This shows the number of studies on tumors that can be treated with combination therapy, with melanoma being the largest. And breast cancer and glioblastoma also have a large number of studies, including the majority of solid tumors. **(C)** Showed the number of studies on tumors treated with immune checkpoint inhibitors combined with OVs, with melanoma being the largest, accounting for 25.6%. **(D)** This shows the number of studies on tumors treated by chemotherapy combined with OVs, with the largest number being abdominal tumors, followed by brain tumors.

Oncolytic viruss can enhance the exposure of tumor antigens, regulate the tumor microenvironment, activate immune cells, and so on through a variety of ways to induce the anti-tumor immune response of the whole-body system to kill tumor cells. The clinical efficacy of first-generation OVs (weakly effective wild-type or naturally occurring variants) was limited, while the new generation of genetically modified OVs has achieved tumor eradication in clinical studies (Bilsland et al., 2016; Russell and Barber, 2018; Jin et al., 2021; Tian et al., 2022). However, current research trends favor the use of combination therapies because monotherapy with OVs faces challenges such as T cell loss due to tumor heterogeneity and the immunosuppressive microenvironment and the difficulty of expression of inhibitory molecules in tumor microenvironment (Zou et al., 2023). Clinical studies have confirmed that the combination of OVs with chemotherapy and the combination of OVs with immunotherapy have achieved remarkable therapeutic effects, surpassing the efficacy of chemotherapy or immunotherapy alone (Tian et al., 2022; Zou et al., 2023). A tumor is not a large number of isolated cancer cells but a complex tissue composed of many different types of cells that perform heterogeneous interactions with each other. This is one reason why a single drug cannot cure cancer completely. Of the 147 clinical registries, 82 (55.8%) were combined studies. Among them, 41% were combined with immune checkpoint inhibitors, and 31% were combined with chemotherapy (Figure 3A). The tumors that can be tested include most solid tumors of the brain, chest, abdomen, and genitals (Figure 3B), which can be treated in combination with other conventional tumor treatment methods. Therefore, OVs can exert greater benefits, promote and increase therapeutic efficacy, and give the oncolytic virus more attention in the international clinical oncology community (Roulstone et al., 2015; Husseini et al., 2017).

The efficacy of immune checkpoint inhibitors has been widely recognized for their efficacy in cancer treatment, but the response rate is not high, and patients with programmed cell death protein 1 (PD-1) resistance make tumor treatment more difficult. Studies have shown that OVs combined with checkpoint inhibitors can significantly improve the objective response rate of various solid tumors, induce a large number of immune cells to infiltrate tumors, and change the tumor microenvironment (Chon et al., 2019), thus enhancing the anti-tumor activity of immunotherapy and the survival rate of human infected with tumor cells. It is an ideal immune platform to enhance immune responses and tumor types in patients with poor response to immune checkpoint suppression, inducing auto proliferation and antitumor activity through a variety of mechanisms. Melanoma was the most tested tumor in combination with immune checkpoint inhibitors, followed by breast cancer (Figure 3C). In 2017, the efficacy of T-VEC combined with PD-1 antibody in the treatment of melanoma was significantly improved (Drescher et al., 2019). The combination of PD-1 inhibitors and oncolytic viruses seems to be the darling of the new age for treating cancer cells, and it is showing signs of potential in clinical trials (Havunen et al., 2021).

Chemotherapy combined with OVs is the most common combination. Chemotherapy is one of the main therapeutic methods that induce DNA damage by inhibiting DNA synthesis, mitosis, and cell division of cancer cells with intracellular toxins and other chemicals. Tumors treated by chemotherapy combined with OVs include celiac tumors, breast cancer, etc. (Figure 3D). Not only does Pelareorep have research reports on the treatment of combined chemotherapy (Mahalingam et al., 2018, 2020) that show that combined chemotherapy is effective, but also other types of oncolytic viruses have research reports that combined chemotherapy is safe (Karapanagiotou et al., 2012; Siurala et al., 2015). Radiotherapy combined with OVs therapy is also a common combination, and some studies show the safety of combined radiotherapy (Markert et al., 2014).

Surgical treatment can generally cure early tumors, but once the tumor metastases, surgery is very difficult, and the tumor cannot be completely cleared. Radiation and chemotherapy have limited effects, serious damage to normal cells and tissues, and great side effects. The overall effectiveness of antibody therapy is not high, and drugs are difficult to break through the barrier and tumor microenvironment. In comparison, OV therapy is safe and reliable, more effective than other immunotherapies, has a simple preparation method, low price, and wide adaptability, and can be used for a variety of cancers. The opportunity for OVs to combine drugs in cancer is huge. It can alter the tumor microenvironment and induce immune cells to infiltrate tumor cells, and various undiscovered anti-tumor properties are being revealed through experiments.

## 3. The process of treating a tumor with an oncolytic virus

### 3.1. Mechanism of action of oncolytic viruses

The oncolytic virus represents a promising immunotherapeutic agent in current cancer therapy. Its anti-tumor activity is mediated by a variety of mechanisms that impact the immune system in tumors.

Natural oncolytic viruses exhibit poor controllability, weak killing ability toward tumor cells, and are easily cleared by the body’s immune system. In contrast, the genetically modified oncolytic virus can be highly expressed in specific areas of the body to avoid triggering the systemic immune response, thereby prolonging the virus’s action time and enhancing its ability to kill tumor cells.

**Figure 4:**
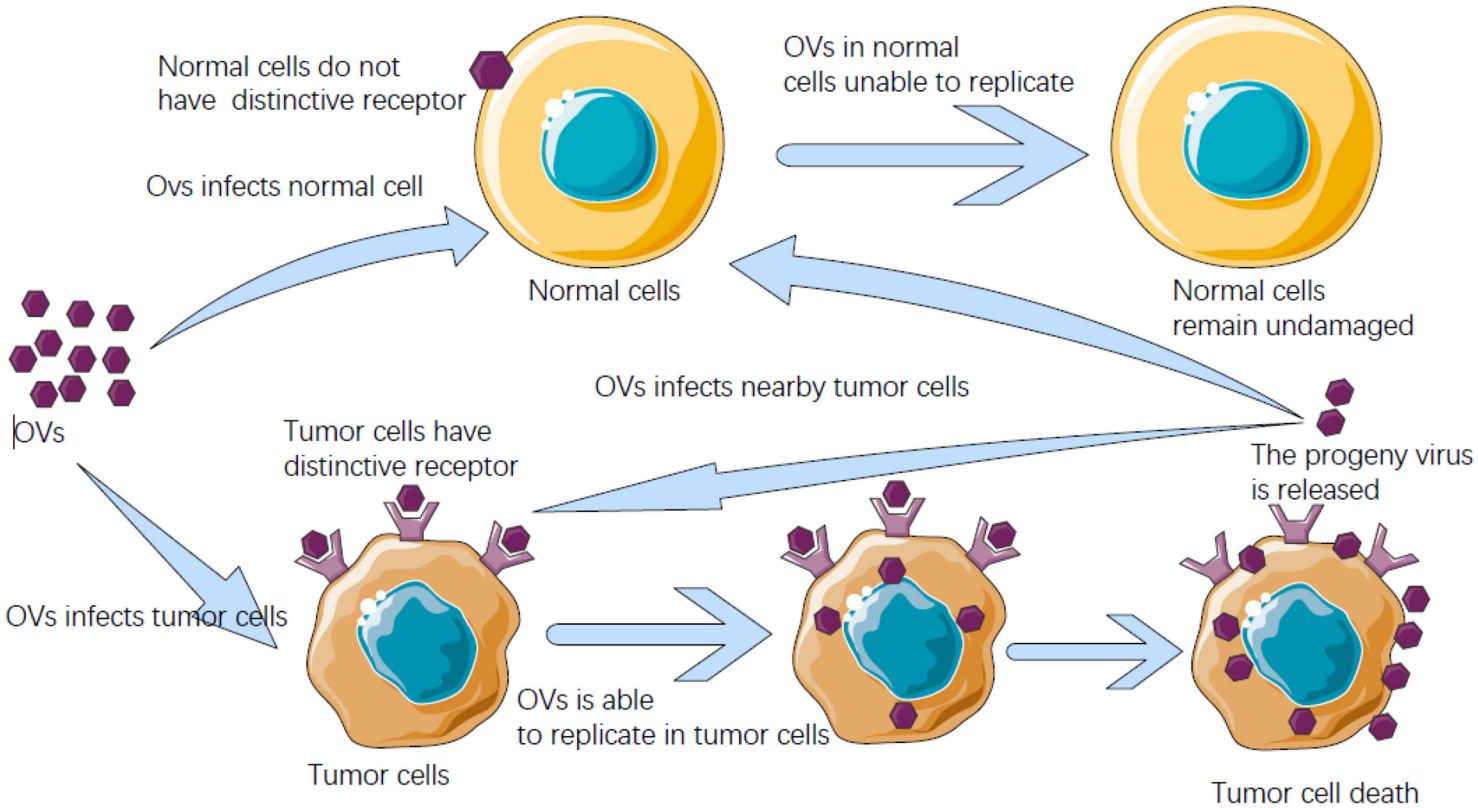
Oncolytic virus (OV) after entering the host can identify receptors on cells, meet with receptors unique to cancer cells can enter the cancer cells, and can breed will kill cancer cells in cancer cells, and cancer cells burst into offspring OVs are released, and can infect nearby cancer cells and kill, and in the normal cells of the human body does not have this kind of peculiar receptor, OVs cannot get into normal cells to multiply.

Tumor cells in the tumor suppressor gene are inactivated or missing after their antiviral infection ability is abated to allow OVs to multiply and ultimately destroy tumor cells in the body. When tumor cells are infected by the virus, they break and die, and then new virus particles are released, further infecting the surrounding tumor cells. This is a kind of antitumor mechanism similar to the chain reaction (Figure 4). OVs mainly eliminate tumor cells through the host’s immune system. That is, OVs not only kill tumor cells on their own but also stimulate the body’s immune system to enhance the effect of treating tumor cells (Kelly and Russell, 2007). This can be applied both systematically and locally and is much better than traditional treatments for cancer cells. OVs fight tumor cells in four ways: oncolysis, antitumor immunity, transgenic expression, and collapse of blood vessels supplying tumor cells (Gujar et al., 2019).

The mechanism of action of OVs can be roughly divided into two parts, one is the direct tumor-killing mechanism mediated by a virus, and the other is the anti-tumor immune response mechanism activated by the body to kill tumor cells.

### 3.2. Oncolytic viruses enter tumor cells

Oncolytic refers to the ability to dissolve tumor cells. To enhance the effectiveness of OVs in targeting tumor cells, research, and development are focused on tumor-specific targeting. Currently, many OVs can mutate to target the surface proteins abnormally expressed by cancer cells, weakening the antiviral ability of tumor cells and providing OVs with an opportunity to enter the tumor cells.

For example, adenoviruses can be designed to target the unique receptors expressed by tumor cells (DeWeese et al., 2001), or they can be inserted into the promoters in tumor cells to utilize the signal pathways to enter tumor cells directly. HSV may enter tumor cells through herpesvirus entry mediator or Nectin (Yu et al., 2005), which are overexpressed in cancer cells, especially melanoma, or it can be designed to bind integrins highly expressed on tumor cells (You et al., 2001). Vaccinia virus can replicate in some cancer cells that overexpress epidermal growth factor receptors by enhancing the induction of RAS signal (Parato et al., 2012). NDV can increase its number in tumor cells by using the B cell lymphoma overexpressed by tumor cells (Mansour et al., 2011). In normal healthy cells, reovirus can enter the cells and start to produce viral RNA, thus activating the double-translated RNA-dependent protein kinase pathway, which is an immune mode that can inhibit protein translation and prevent virus transmission (Bischoff and Samuel, 1989). However, the double-translated RNA-dependent protein kinase pathway will not be activated in cancer cells, causing the virus to infect tumor cells and eventually leading to the splitting of cancer cells (Strong et al., 1998).

At the same time, many OVs can rapidly replicate by using the defect of the antiviral mechanism of tumor cells in type I interferon signal (Stojdl et al., 2000; Wilden et al., 2009). The measles virus can use the surface receptor CD46, a factor that prevents cells from being destroyed and is overexpressed by cancer cells through the complement pathway of the immune system (Dorig et al., 1993; Anderson et al., 2004), or it can be designed to recognize carcinoembryonic antigen and directly enter tumor cells (Hammond et al., 2001).

### 3.3. Tumor-killing mechanism mediated by an oncolytic virus

Here are some ways in which OVs can kill tumors.

Firstly, through virus-mediated cytotoxic killing, OVs use the energy and raw materials of tumor cells as a breeding site for their proliferation. The virus can inhibit the growth of tumor cells and use the cell as a factory for replication and lysis of tumor cells. After the release of the progeny virus, it continues to infect neighboring tumor cells, creating a cycle that continues until the immune response weakens the replication of the virus or the tumor cells in the host are depleted (Hamid et al., 2017). Because the antiviral response of tumor cells has certain defects, oncolytic viruses will preferentially replicate in tumor cells, while in normal cells, viruses can hardly replicate due to the presence of interferon and related factors. Additionally, some viruses can produce substances with certain cytotoxicity and oncolytic activity. For example, proteins encoded by the E3 region of Ad can directly mediate the lysis of tumor cells (Suzuki et al., 2002).

**Figure 5:**
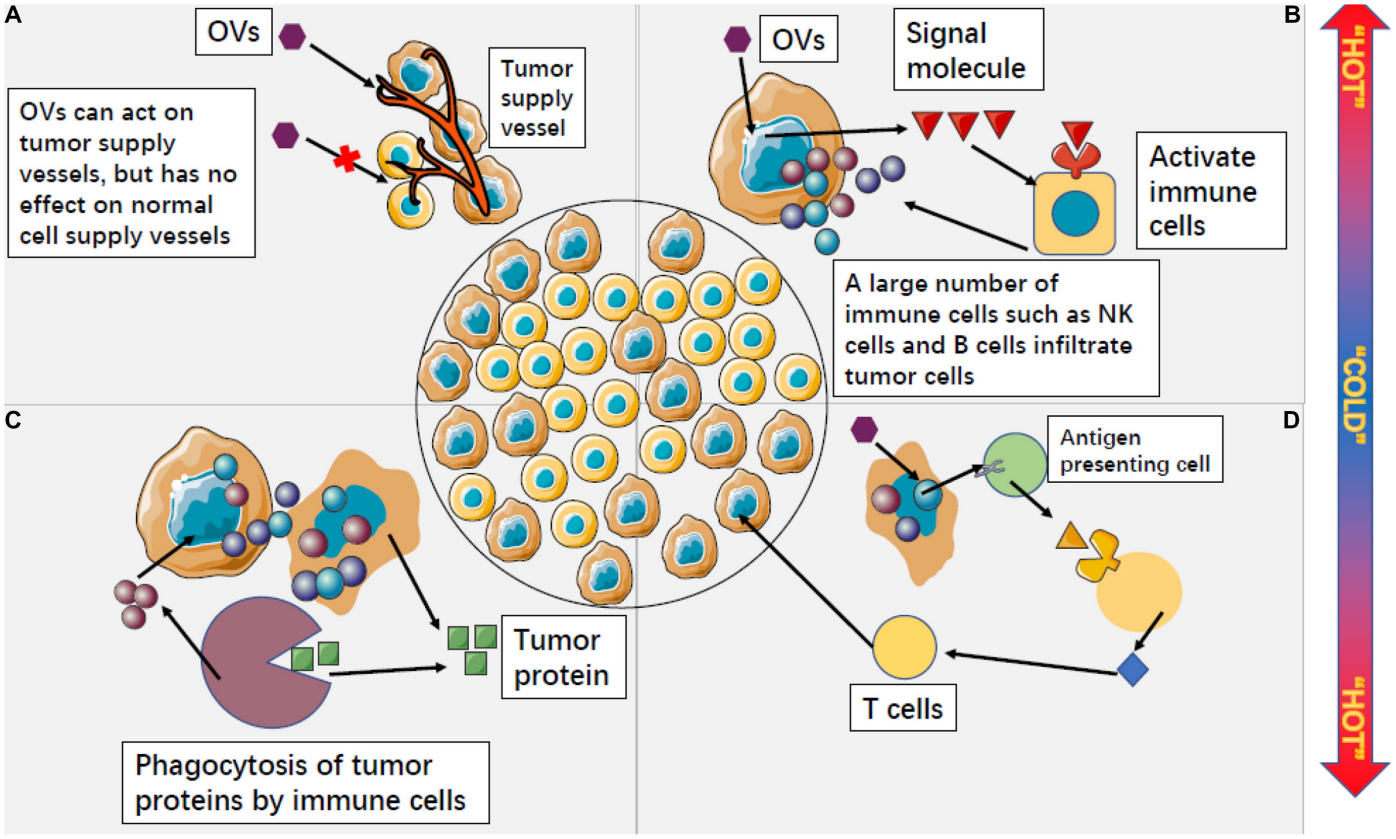
Oncolytic viruses can change the microenvironment of tumor cells from “cold” to “hot.” **(A)** OVS can specifically attack the supply vessels of tumor cells, making tumor cells have no nutritional supply to kill tumor cells, while OVS have no response to the supply vessels of normal cells. **(B)** After OVs act on tumor cells, tumor cells can release a large number of signal molecules to activate non-specific immune cells outside tumor cells. A large number of activated immune cells infiltrate tumor cells and accelerate the process of killing tumor cells. **(C)** Cleaved tumor cells will release a large amount of tumor protein, but non-specific immune cells can use this tumor protein to continuously play an immune role and form a long-term anti-tumor effect. **(D)** OV activates specific antigens in tumor cells, which can be expressed by antigen-presenting cells and induce the killing effect of T cells.

Secondly, OVs can indirectly kill tumors by destroying the vascular system of tumor cells. Tumor growth depends on the nutrients provided by the tumor vascular system, so the destruction of the tumor vascular system can effectively inhibit tumor growth (Figure 5A). Angiogenesis is one of the markers of malignant tumor cells, and research shows that oncolytic viruses can specifically infect and destroy tumor vascular endothelial cells and stromal cells, as well as destroy tumor blood vessels by promoting the production of endostatin and angiostatin (Hutzen et al., 2014). For example, the oncolytic vaccinia virus has been proven to inhibit the formation of vascular endothelial cells of tumor cells, thereby damaging the tumor vascular system and inhibiting the formation of new blood vessels. This reduces the blood flow to supply tumor cells and ultimately limits the growth and development of tumors (Goradel et al., 2017; Hashemi Goradel et al., 2018). VSV can directly infect and damage tumor blood vessels in the body without affecting normal blood vessels (Breitbach et al., 2011b).

### 3.4. Mechanism of anti-tumor immune response induced by oncolytic viruses

The tumor-bearing host cannot make an effective immune response to prevent tumor growth. The inhibition state of the tumor microenvironment is due to the inactivation of cytotoxic T lymphocytes (Jiang and Shapiro, 2014) or active inhibitory T cells (Postow et al., 2018), leading to tumor immune escape.

Oncolytic viruss have a strong stimulation effect on the immune cells in the existing tumor tissues and can greatly change the tumor microenvironment, changing the tumor from cold to hot. By doing so, OVs can convert the immunosuppressive tumor microenvironment into an immunostimulatory one, thereby promoting anti-tumor immune responses (Chaurasiya et al., 2018; Sivanandam et al., 2019). Tumor cells infected with OVs can express some signaling molecules that induce immune cells outside the tumor to infiltrate the tumor in large numbers (Figure 5B; Ribas et al., 2017). Activated nonspecific immune cells kill and engulf other tumor cells, and lysed tumor cells release large amounts of tumor proteins that can be engulfed by innate immune cells and produce long-term adaptive immune responses against tumor-specific antigens (Figure 5C; Del Papa et al., 2019; Chouljenko et al., 2020; Kdimati et al., 2021). Some tumor-specific antigens can also be expressed by antigen-presenting cells, inducing T cells to attack uninfected tumor cells. This process is similar to a vaccine, but it occurs within tumor tissues and ultimately induces the killing effect of CD8 T cells, effectively preventing tumor recurrence and metastasis (Figure 5D; Macleod et al., 2014; Eissa et al., 2021; Yamashita et al., 2021).

Type I interferon can not only promote the immune response of clearing a virus but also reduce cell proliferation and activate apoptosis-promoting protein p53 (Takaoka et al., 2003). Therefore, OVs are more specific for tumor cell environment or an environment with limited type I interferon response.

The anti-tumor effect of the oncolytic virus seems to have immune memory. Some experiments have shown that the efficacy of OV injected into the mice immunized with OV in advance is better than that of the mice immunized without OV (Zhu et al., 2007). At the same time, some studies have shown that repeated intratumoral injection of OV can prolong its anti-tumor effect and induce lasting anti-tumor memory (Ghonime et al., 2018). It is possible that vaccines that have been injected as OV, such as the measles virus, may also act on anti-tumor (Qi et al., 2018; Leb-Reichl et al., 2021), but may also inhibit the anti-tumor effect (Laksono et al., 2018; Rudak et al., 2021).

Although OVs infection can trigger anti-tumor immunity, the immune system can also inhibit the virus, leading to reduced efficacy. OVs may cause the human body to acquire live viruses with acute toxicity and, in some cases, may cause latent infections and chronic diseases. This pathogenicity largely depends on the type of virus, virulence, immune destructiveness, and the host’s immune response. At present, no serious adverse events have been reported in the clinical trials, but the follow-up of the experimental patients is incomplete. Most OVs currently use toxicity reduction to prevent acute and chronic poisoning (Mejias-Perez et al., 2018; Nakatake et al., 2019).

As a pathogen, the pathogenicity of the oncolytic virus cannot be ignored. Some viruses may damage normal tissues during tumor lysis, so the biological safety of oncolytic virus therapy deserves further research, especially for people with low immunity. After chemotherapy, radiotherapy, and other traditional treatment schemes for cancer patients, bone marrow is usually damaged, and the body cannot produce a normal number of red blood cells, white blood cells, and platelets. When the body does not have enough white blood cells, oncolytic virus therapy is fatal (Perkins, 2002). Although some oncolytic viruses are not toxic, the body does not have enough immunity to resist the oncolytic virus, which will lead to toxic reactions. T-VEC guidelines clearly state that people with low immunity or who are pregnant should avoid using T-VECs. At present, the potential safety problems and long-term adverse events of the oncolytic virus are still unclear (Li et al., 2020).

## 4. Problems and challenges of oncolytic virus therapy for tumors

Although OVs can respond well to tumor cells, they still face several challenges. One challenge is how OV manipulates the host’s immune system to promote anti-tumor immune responses while limiting antiviral immune responses and virus clearance with minimal damage (Tan et al., 2020; Wu et al., 2021). Systemic drug administration has higher value and application prospects and is expected to cure metastatic tumors or blood tumors (Zhang et al., 2020; Tang et al., 2021; Xie et al., 2021). However, the therapeutic effect of systemic drug delivery is not ideal, and researchers need to spend a lot of energy to limit the antiviral immune response of systemic drug delivery. Using cell vectors to deliver oncolytic viruses to tumors without damaging surrounding tissues is one strategy to overcome this challenge.

Another huge challenge is to select the right patients to use OVs for clinical treatment. Tumor patients receiving OV treatment may have already undergone multiple conventional tumor treatments, which may have damaged their immune systems. One of the mechanisms underlying the efficacy of oncolytic viruses is their ability to trigger immune responses in cancer patients (Chaurasiya et al., 2021). Therefore, future research can focus on how to assess the immune system function of tumor patients and determine whether they should receive oncolytic virus therapy based on these evaluations. And selecting appropriate biomarkers may effectively improve the treatment of OVs.

As a live virus, OV can activate the immune system of the body. How to inhibit antiviral immunity and enhance anti-tumor immunity is a new challenge in the treatment process. Moreover, current research shows that OV needs to be combined with some tumor therapies to achieve the maximum therapeutic effect. In the future, the application value of monotherapy remains to be explored.

## 5. Conclusion and prospects

Although OVs therapy has made great progress, there are many obstacles in the process of treatment. With the continuous development of science and technology, as well as people’s in-depth research on the mechanism of action of oncolytic virus, the mode of administration, and the pathogenesis of tumors, the use of an oncolytic virus, combined with other therapies to treat tumors and other methods provide a new direction for targeted treatment of tumors. Oncolytic viruses will become the main force in the treatment of tumors in the future.

## Author contributions

XL, XZ, and TT conceived the work. XZ, CF, ZX, MC, and ZL wrote and drafted the manuscript. XZ, ZX, TT, and XL discussed and edited the manuscript. All authors contributed to the article and approved the submitted version.

## Funding

This work was supported partly by The Wu Jieping Medical Foundation Clinical Research Special Grant—Application of High-Throughput Rapid Detection Technology for Pathogenic Pathogens (No. 320.6750.2021-06-29).

## Conflict of interest

The authors declare that the research was conducted in the absence of any commercial or financial relationships that could be construed as a potential conflict of interest.

## Publisher’s note

All claims expressed in this article are solely those of the authors and do not necessarily represent those of their affiliated organizations, or those of the publisher, the editors and the reviewers. Any product that may be evaluated in this article, or claim that may be made by its manufacturer, is not guaranteed or endorsed by the publisher.
